# Ca^2+^/H^+^ exchange, lumenal Ca^2+^ release and Ca^2+^/ATP coupling ratios in the sarcoplasmic reticulum ATPase

**DOI:** 10.1007/s12079-013-0213-7

**Published:** 2013-12-04

**Authors:** Giuseppe Inesi, Francesco Tadini-Buoninsegni

**Affiliations:** 1 California Pacific Medical Center Research Institute, 475 Brannan Street, San Francisco, CA 94107 USA; 2 Department of Chemistry “Ugo Schiff,”, University of Florence, 50019 Sesto, Fiorentino Italy

**Keywords:** SERCA Ca^2+^ATPase, Ca^2+^/ATP coupling ratios, Ca^2+^/H^+^ exchange, Ca^2+^ signaling, Sarcolipin, Phospholamban, Thermogenesis

## Abstract

The Ca^2+^ transport ATPase (SERCA) of sarcoplasmic reticulum (SR) plays an important role in muscle cytosolic signaling, as it stores Ca^2+^ in intracellular membrane bound compartments, thereby lowering cytosolic Ca^2+^ to induce relaxation. The stored Ca^2+^ is in turn released upon membrane excitation to trigger muscle contraction. SERCA is activated by high affinity binding of cytosolic Ca^2+^, whereupon ATP is utilized by formation of a phosphoenzyme intermediate, which undergoes protein conformational transitions yielding reduced affinity and vectorial translocation of bound Ca^2+^. We review here biochemical and biophysical evidence demonstrating that release of bound Ca^2+^ into the lumen of SR requires Ca^2+^/H^+^ exchange at the low affinity Ca^2+^ sites. Rise of lumenal Ca^2+^ above its dissociation constant from low affinity sites, or reduction of the H^+^ concentration by high pH, prevent Ca^2+^/H^+^ exchange. Under these conditions Ca^2+^ release into the lumen of SR is bypassed, and hydrolytic cleavage of phosphoenzyme may yield uncoupled ATPase cycles. We clarify how such Ca^2+^pump slippage does not occur within the time length of muscle twitches, but under special conditions and in special cells may contribute to thermogenesis.

## Introduction

Sarcoplasmic reticulum (SR) membrane vesicles, originally referred to as “relaxing factor”, were first isolated from skeletal muscle by Ebashi and Lipmann (1962), and Hasselbach and Makinose (1962), and were shown to contain a P–type ATPase (SERCA1 isoform) sustaining Ca^2+^ transport. In muscle cells, this transport activity plays an important role in lowering cytosolic Ca^2+^ as required for relaxation of contractile elements, and storing transported Ca^2+^ in the lumen of SR for subsequent release and contractile activation (Carafoli 2002; Clapham 2007). General information on SERCA1 catalytic function and molecular structure is given in several reviews (de Meis and Vianna 1979; Inesi et al. 1990; Andersen and Vilsen 1995; Toyoshima 2008; Møller et al. 2010).

**Fig. 1:**
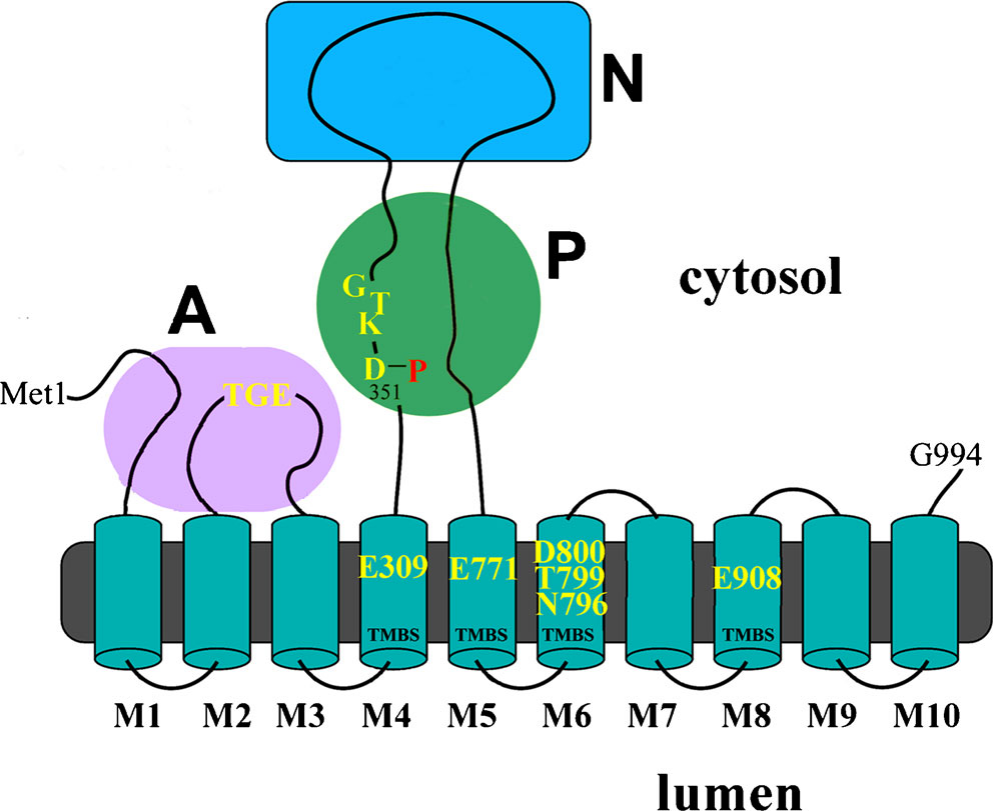
Two-dimensional folding model of the SERCA1 sequence. The diagram shows ten transmembrane segments (M1 to M10) including six residues (Glu-309, Glu-771, Asn-796, Thr-799, Asp-800 and Glu-908) contributing oxygen atoms for calcium binding, enzyme activation, and transport. The extramembranous headpiece comprises: a nucleotide binding domain (N); the P domain, with several residues conserved in P-type ATPases, including Asp-351 (in red) that undergoes phosphorylation to form the catalytic phosphoenzyme intermediate (EP); and the A domain with the Thr-Gly-Glu conserved sequence involved in catalytic assistance of EP hydrolytic cleavage

SERCA1 is a 996 amino acid membrane bound protein (MacLennan et al. 1985) comprising ten transmembrane helical segments, and a globular headpiece that protrudes from the cytosolic side of the membrane and includes three distinct domains (A, N and P). Catalytic activation follows high affinity binding of cytosolic Ca^2+^ within the transmembrane region of the enzyme (Fig. 1). Activation is followed by utilization of ATP bound to the N domain, and formation of phosphorylated enzyme intermediate by transfer of the ATP γ-phosphate to an aspartyl residue (Asp-351) in the P domain. Conformational transition of the phosphoenzyme then promotes vectorial translocation of bound Ca^2+^ and release of Ca^2+^ into the lumen of SR. Finally, the phosphoenzyme undergoes hydrolytic cleavage with catalytic assistance by an A domain critical sequence (Thr-Gly-Glu), leading to a new cycle.

## Ca^2+^/ATP coupling ratios

**Fig. 2:**
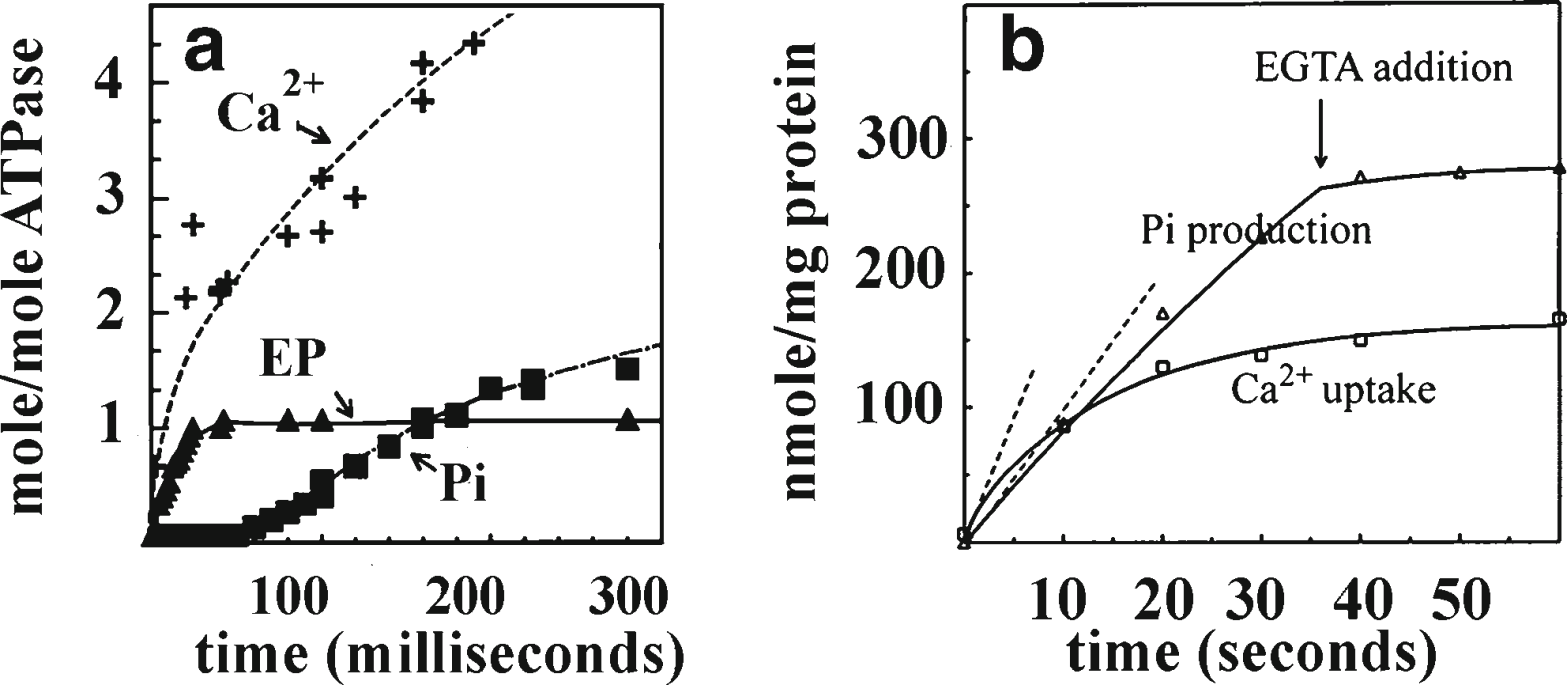
Pre-steady state measurements of ATPase activity and Ca^2±^ transport by native SR vesicles obtained from skeletal muscle. **a** Initial phosphoenzyme formation and Ca^2+^ occlusion (2Ca^2+^/1EP) are observed within the first cycle following addition of ATP. Ca^2+^ uptake and Pi production rates follow with molar ratios of 2:1. Time resolution in the millisecond time scale was obtained with rapid mixing instruments. **b** Pre-steady state experiments extended to the second time scale, show that the initial rates of Ca^2+^ uptake and Pi production begin with a ratio of 2:1, but the Ca^2+^ uptake rate undergoes saturation, while uncoupled ATPase activity continues as long as the medium Ca^2+^ is maintained above the ATPase activation level. Uncoupled ATPase ceases if EGTA is added to chelate medium Ca^2+^. Reaction mixtures contained 20–50 μg SR protein/ml, 10 mM PIPES, pH 7.0, 100 mM KCl, 5 mM MgSO_4_, 0.2 mM CaCl_2_ and 0.2 mM EGTA. Radioactive tracers added according to the experimental schedule. Reaction started with 100 mM ATP and stopped by acid quenching. 1 mM EGTA added when indicated. Temperature 25 °C. Derived from Inesi et al. (1988) and Yu and Inesi (1995)

Cooperative binding of 2 Ca^2+^ per ATPase (Inesi et al. 1980) implies transport of 2 Ca^2+^ per catalytic cycle, if both bound Ca^2+^ are translocated with maximal efficiency. Ratios of 2 Ca^2+^ per ATP were in fact observed under conditions permitting free Ca^2+^ to remain low in the lumen of the vesicles: (*a*) steady state experiments in which oxalate is used for complexation of lumenal Ca^2+^ (Martonosi and Feretos 1964) and (*b*) pre-steady state experiments in which lumenal Ca^2+^ has yet to rise (Fig. 2a; Inesi et al. 1988). On the other hand, Ca^2+^/ATP ratios lower than 2 have been observed with native SR vesicles as well as reconstituted systems (Yu and Inesi 1995), under conditions permitting lumenal Ca^2+^ to rise (mM) while Ca^2+^ in the outer medium remains sufficiently high (μM) for ATPase activation (Fig. 2b). Under these conditions, the lumenal Ca^2+^ concentration is higher than the dissociation constant of Ca^2+^ from the lumenal sites, and therefore the phosphoenyme bypasses the Ca^2+^ release step and proceeds to hydrolytic cleavage of Pi, with consequent reduction of the Ca^2+^/ATP transport ratio. Uncoupled ATPase subsides if EGTA is added to the outer medium to reduce free Ca^2+^ below the ATPase activating level (Fig. 2b).

A variable stoichiometric ratio (i.e., Ca^2+^/ATP) of active transport may be considered to be an intrinsic feature of the pump, if the ATPase reaction sequence allows an alternate pathway leading to hydrolytic cleavage of P_i_ without vectorial displacement of Ca^2+^ (Johnson et al. 1985; Inesi and de Meis 1989). The importance of this phenomenon, referred to as *slippage of the pump*, is related to heat production and thermogenesis, when the free energy derived from ATP hydrolysis is not utilized for active transport (de Meis et al. 1997; de Meis 2001; de Meis et al. 2005).

## Ca^2+^/H^+^ exchange at the lumenal gate

**Fig. 3:**
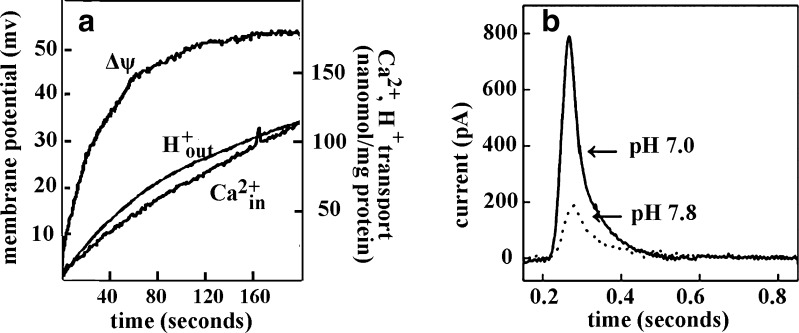
**a** ATP-dependent Ca^2±^ uptake, H^±^ countertransport, and development of transmembrane electrical potential in reconstituted SERCA1 proteoliposomes. Proteoliposomes prepared at pH 7.0 were diluted (5.0 μg protein/ml) in a medium (pH 7.0) containing 10 mM PIPES, 100 mM K_2_SO_4_, 5 mM MgSO_4_, 50 μM CaCl_2_, and 50 μM arsenazo III, or 200 μM lumenal pyranine, or 1 μM oxonol VI. The reaction was started at 11 °C by the addition of 0.2 mM ATP and followed by differential absorption spectrometry. **b** Charge measurements on native SR Ca^2±^ATPase (SERCA1) adsorbed on a solid supported membrane (SSM). The current transients were obtained after rapid delivery of 100 μM ATP to ATPase preincubated with 10 μM free Ca^2+^ and 100 mM KCl, at pH 7 (solid line) or pH 7.8 (dotted line). Derived from Yu et al. (1994) and Lewis et al. (2012)

Exchange of Ca^2+^ with H^+^ upon vectorial translocation is a specific feature of the Ca^2+^ ATPase (Lewis et al. 2012), facilitating lumenal Ca^2+^ release (Yu et al. 1994; Bublitz et al. 2013). Evidence of Ca^2+^/H^+^ exchange, H^+^ counter transport (Chiesi and Inesi 1980; Yamaguchi and Kanazawa 1985; Ueno and Sekine 1981) and electrogenicity (Morimoto and Kasai 1986; Cornelius and Møller 1991; Obara et al. 2005) in the operation of the Ca^2+^ ATPase was obtained with vesicular fragments of SR membrane and with ATPase reconstituted in phospholipids vesicles lacking non specific H^+^ or Ca^2+^ channels. It is shown in Fig. 3a that the molar ratio of Ca^2+^/H^+^ counter transport is 1 when the lumenal and medium pH is near neutrality. However, a higher number of acidic residues involved in Ca^2+^ binding (Glu-771, Asp-800, Glu-309, Glu-908) is likely to participate in Ca^2+^/H+ exchange (Bublitz et al. 2013; Obara et al. 2005), even though only one H^+^ per Ca^2+^ may actually be counter transported. In this case, the remaining H^+^ undergo lumenal dissociation. The Ca^2+^/H^+^ exchange is facilitated by acidic residues pK changes, as the phosphoenzyme undergoes its catalytic transition (Yu et al. 1994).

Further evidence for Ca^2+^/H^+^ exchange is provided by measurements of charge transfer upon addition of Ca^2+^ or ATP to microsomal vesicles adsorbed on a solid supported membrane (SSM) (Tadini-Buoninsegni et al. 2004, 2006, 2010). Related electrogenic events are recorded as current transients due to flow of electrons along the external circuit toward the electrode surface, as required to compensate for the potential difference across the vesicular membrane produced by displacement of positive charge upon vectorial translocation in the direction of the SSM electrode. In fact, when ATP is added to the membrane bound ATPase adsorbed on the SSM in the presence of saturating Ca^2+^, a current transient is observed due to vectorial translocation and dissociation of bound Ca^2+^ in the direction of the SSM electrode after phosphoenzyme formation by utilization of ATP (Tadini-Buoninsegni et al. 2004, 2006). The electrical current recorded by the SSM method is a measure of the rate of change of the transmembrane potential and is not sensitive to stationary currents. Therefore, only the electrogenic signal generated within the first cycle is observed, whereas steady state events after the first cycle are not detected. It is shown in Fig. 3b that the net charge produced by ATP addition at neutral pH decreases significantly if ATP addition is performed at alkaline pH. This indicates that when lack of H^+^ limits H^+^/Ca^2+^ exchange (i.e. alkaline pH), vectorial translocation of bound Ca^2+^ in the direction of the SSM electrode is prevented, even though K^+^ is present in high concentration and may neutralize acidic residues at alkaline pH. This indicates a requirement for specific H^+^ binding at the Ca^2+^ sites, in order to obtain Ca^2+^ release.

The specific relevance of ATP dependent charge transfer is demonstrated by interference of mutations (Asp-351 to Asn) preventing phosphoenzyme formation (Tadini-Buoninsegni et al. 2006). Furthermore, cation/H^+^ exchange at the transport sites following phosphoenzyme formation occurs in Ca^2+^ ATPases, but does not occur in copper ATPases (Lewis et al. 2012).

**Fig. 4:**
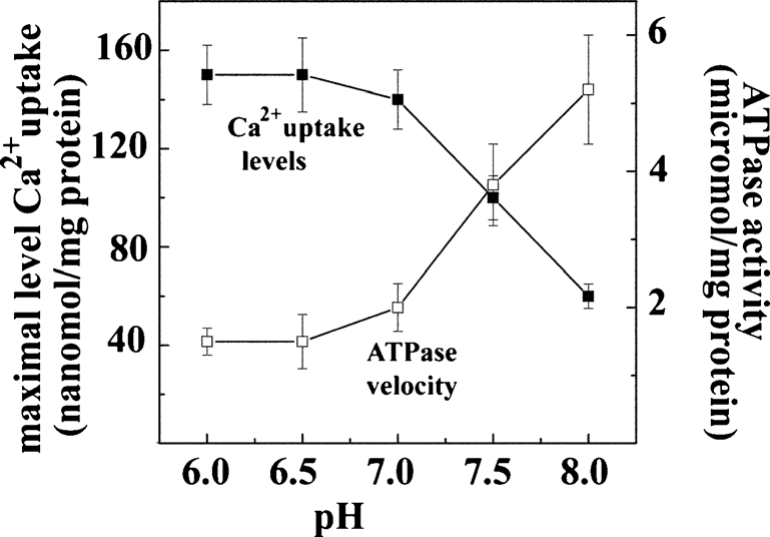
Maximal levels of Ca^2±^ uptake and rates of ATPase activity in the absence of oxalate as a function of pH. Experiments performed as in Fig. 2B, except for pH regulation with 50 mM MES or HEPES buffer. The reaction was started by addition of 1 mM ATP, at 25 °C. Original data

The importance of Ca^2+^/H^+^ exchange in determining release of bound Ca^2+^ from the phosphoenzyme can be also demonstrated in steady state experiments. It is shown in Fig. 4 that the maximal levels of accumulated Ca^2+^ are significantly reduced if the pH is raised above 7 (consider that the physiological intracellular pH is 6.8, while the extracellular pH is 7.4)). This indicates that if exchange is limited due to low H^+^ concentration, Ca^2+^ is less likely to dissociate from the phosphoenzyme. On the other hand, while Ca^2+^ translocation is reduced, steady state ATPase activity is increased as the pH is raised, and continues after maximal levels of Ca^2+^ uptake are reached. It is apparent that alkaline pH reduces Ca^2+^/H^+^ exchange and dissociation of bound Ca^2+^, whereby the phosphoenyme bypasses the Ca^2+^ release step and proceeds to hydrolytic cleavage of Pi. Therefore, reduction of the Ca^2+^/ATP transport ratio can be produced either by a high Ca^2+^ concentration or a low H^+^ concentration in the lumen of the vesicles.

**Scheme 1:**
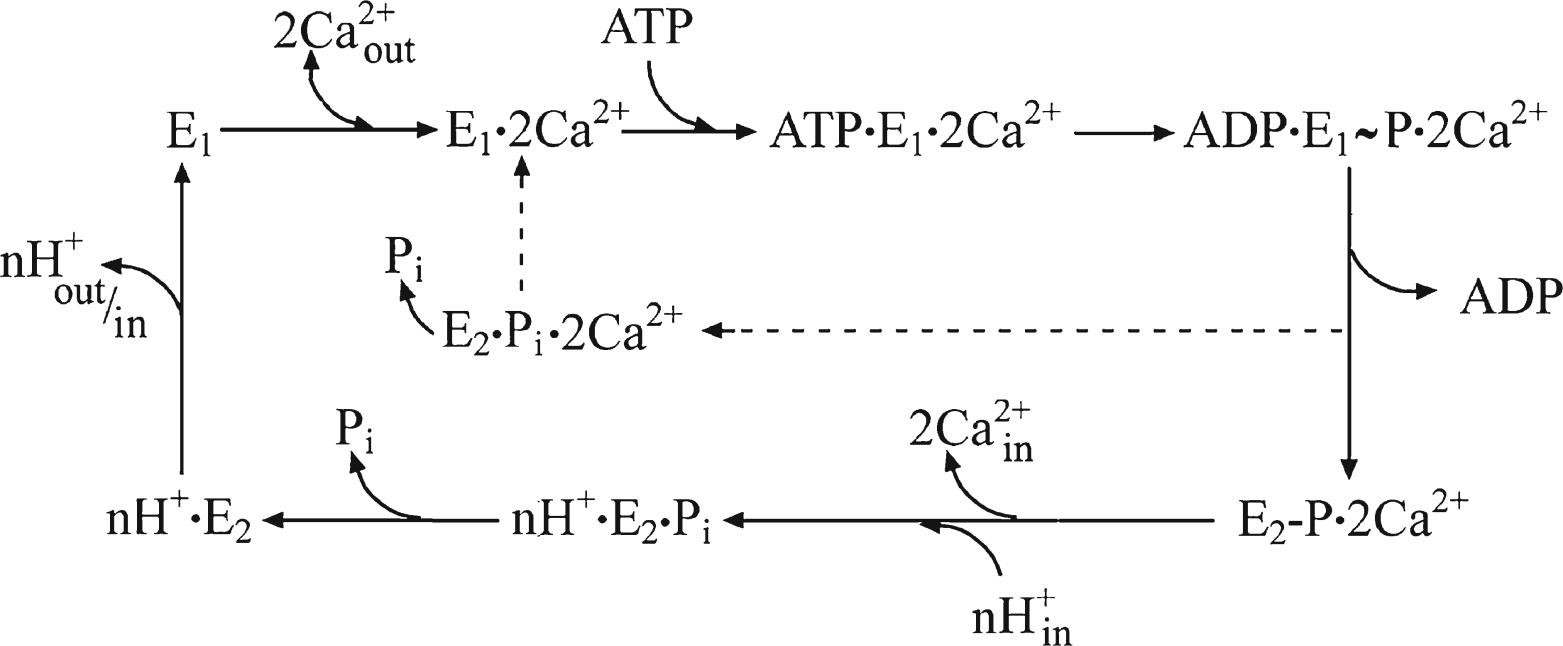
Diagram outlining the sequential reactions on a SERCA catalytic and transport cycle as explained in the text above. The solid lines indicate the optimal pathway of a well coupled ATP utilization and net Ca2+. The dotted lines indicate a short cut of the enyme cycle, whereby ATP utilization is uncoupled from net Ca2+ transport, as explained in the text above

A diagram of the sequential steps in the ATPase mechanism, derived from the original reaction diagram of de Meis and Vianna (1979) and modified to show Ca^2+^/H^+^ exchange and a pathway for slippage of the Ca^2+^ pump, is given in Scheme 1.

In the diagram on Scheme 1, solid lines indicate the optimal pathway, beginning with enzyme activation by high affinity and cooperative binding of two Ca^2+^, yielding E_1_ · 2Ca^2+^. Utilization of ATP yields ADP · E_1_ **~** P · 2Ca^2+^, followed by release of ADP and utilization of the phosphorylation potential to change vectorial orientation and affinity of the Ca^2+^ sites. Bound Ca^2+^ is then released into the lumenal medium in exchange for H^+^. Hydrolytic cleavage of nH^+^ · E_2_-P and transition of nH^+^ · E_2_ to E_1_ finally yields closure of the lumenal gate and exposure of the Ca^2+^ sites to the cytosolic medium. Formation of E_1_ · 2Ca^2+^ then starts a new cycle.

**Fig. 5:**
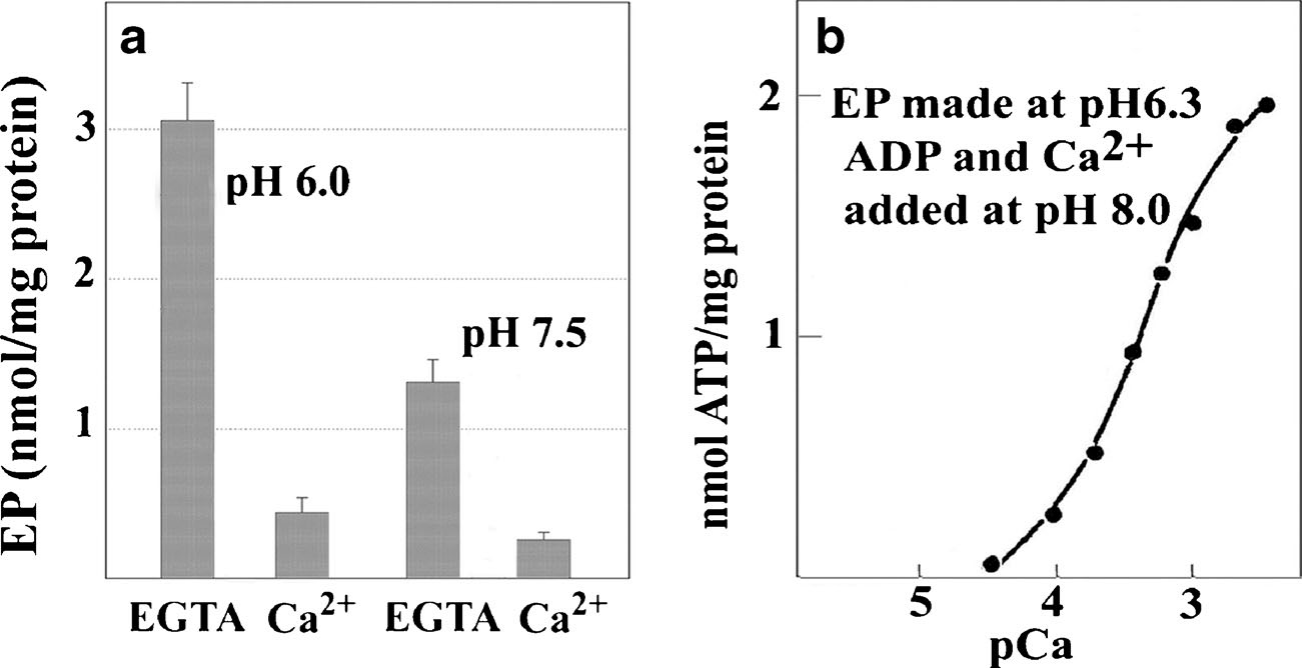
**a** Equilibrium levels of phosphoenzyme obtained through utilization of Pi by SERCA1 at acid or alkaline pH, in the absence or presence of Ca^2±^. Reaction medium: 50 mM MES (pH 6.0) or HEPES (pH 7.5), 20 % Me_2_SO_4_, 10 mM MgCl_2_, 100 mM KCl, 2 mM EGTA or 1 mM CaCl_2_ (in the absence of EGTA), and 50 μg protein/ml. The reaction was started by the addition of 50 μM [^32^P]Pi. The samples were acid quenched after 2 min incubation at 30 °C, and processed by electrophoresis for determination of radioactive ATPase protein. **b** Ca^2±^ concentration and pH dependence of phosphoryl transfer from phosphoenzyme to ADP, to yield ATP. Phosphoenzyme was obtained by incubating 3.0 mg of protein/ml in 60 mM Tris-maleate, pH 6.3, 4 mM [^32^P]Pi, 20 mM MgC1_2_, 0.5 mM EGTA. Following a 2 min incubation at 30 °C, the reaction mixture was diluted 10-fold with 60 mM Tris-maleate (pH 8.0), 1 mM ADP, CaC1_2_ and EGTA to yield free Ca^2+^ as indicated. The samples were acid quenched after 5 min incubation and processed for determination of ATP. Derived from Lewis et al. (2012) and de Meis and Inesi (1982)

The dotted lines in Scheme 1 indicate that if lumenal Ca^2+^ is higher than its dissociation constant, or lumenal H^+^ is too low to sustain exchange, Ca^2+^ release and formation of nH^+^ · E_2_ are prevented. Interference with completion of the ATPase cycle would then cause reversal to E_1_ **~** P · 2Ca^2+^ (see below Fig. 5b), whereby phosphorylation potential leads directly to hydrolytic cleavage, rather than utilization for active transport. This is rendered possible as low concentration of ADP prevents its re-binding, and the remaining proximity of the A domain to the phosphorylation site allows catalytic assistance by the critical Thr-Gly-Glu sequence.

Strong evidence for the role of protons and the nH^+^ · E2 state is provided with experiments of enzyme phosphorylation by utilization of Pi (Masuda and de Meis 1973). It is shown in Fig. 5a that this reaction (i.e., reverse reaction of phosphoenzyme hydrolytic cleavage) is enhanced by acid pH, and is inhibited by alkaline pH and Ca^2+^. This indicates a requirement for proton occupancy of acidic residues involved in Ca^2+^ binding (i.e., nH^+^ · E_2_). On the other hand (Fig. 5b), further reversal of the cycle upon addition of ADP to form ATP, requires a switch to alkaline pH and addition of mM Ca^2+^, in order to replace protons with Ca^2+^ on the low affinity binding sites (i.e. transition of nH^+^ · E_2_-P to E_2_-P · 2Ca^2+^ and ADP · E_1_ ~ P · 2Ca^2+^).

High resolution crystal structures of most states (or their analogs) comprising the ATPase reaction sequence have been obtained, and are described in detailed reviews (Toyoshima 2008; Møller et al. 2010; Toyoshima and Inesi 2004). Comparison of these structures reveals rearrangements of transmembrane helices upon Ca^2+^ binding, phosphoenzyme formation, occlusion and then dissociation of bound Ca^2+^, which are mechanically linked to specific bending and rotation patterns of each headpiece domain. These movements provide an explanation for the long range linkage of phosphorylation and Ca^2+^ binding domains, including the roles of critical amino acids in substrate binding, catalytic reactions, and Ca^2+^ transport. They also demonstrate that the states included in the reaction diagram are structurally distinct, and possess specific features that are functionally relevant. It is of interest that movement of M1-M2 causes displacement of membrane helix M4L from M5 and M6, thereby opening the lumenal gate and allowing outflow of Ca^2+^, following Ca^2+^/H^+^ exchange. The lumenal gate is then closed upon cleavage of phosphate and dissociation of H^+^, when reverse rotation of the A domain is accompanied by upward displacement of M4L and reduction of the space between the M4 and M6 helices.

## Effects of accessory polypeptides

Several studies have demonstrated that sarcolipin (SLN), a 31 amino acid polypeptide (Odermatt et al. 1997; Odermatt et al. 1998) is constitutively bound to the Ca^2+^ ATPase (SERCA1) of (at least human and rabbit) fast twitch skeletal muscle, and produces uncoupling of ATP utilization and Ca^2+^ transport, with a consequent thermogenic effect (Mall et al. 2006; Bal et al. 2012). However, these studies were performed by genetic manipulations and reconstitution procedures, which may not apply to the physiologic signaling mechanism of a fast twitch in native muscles, but rather reflect phenomena occurring under special circumstance and/or in other tissues (see below). In fact, other studies have shown that incorporation of SLN into proteoliposomes with SERCA simply results in a lower apparent affinity for calcium and a lower turnover rate (Gorski et al. 2013).

**Fig. 6:**
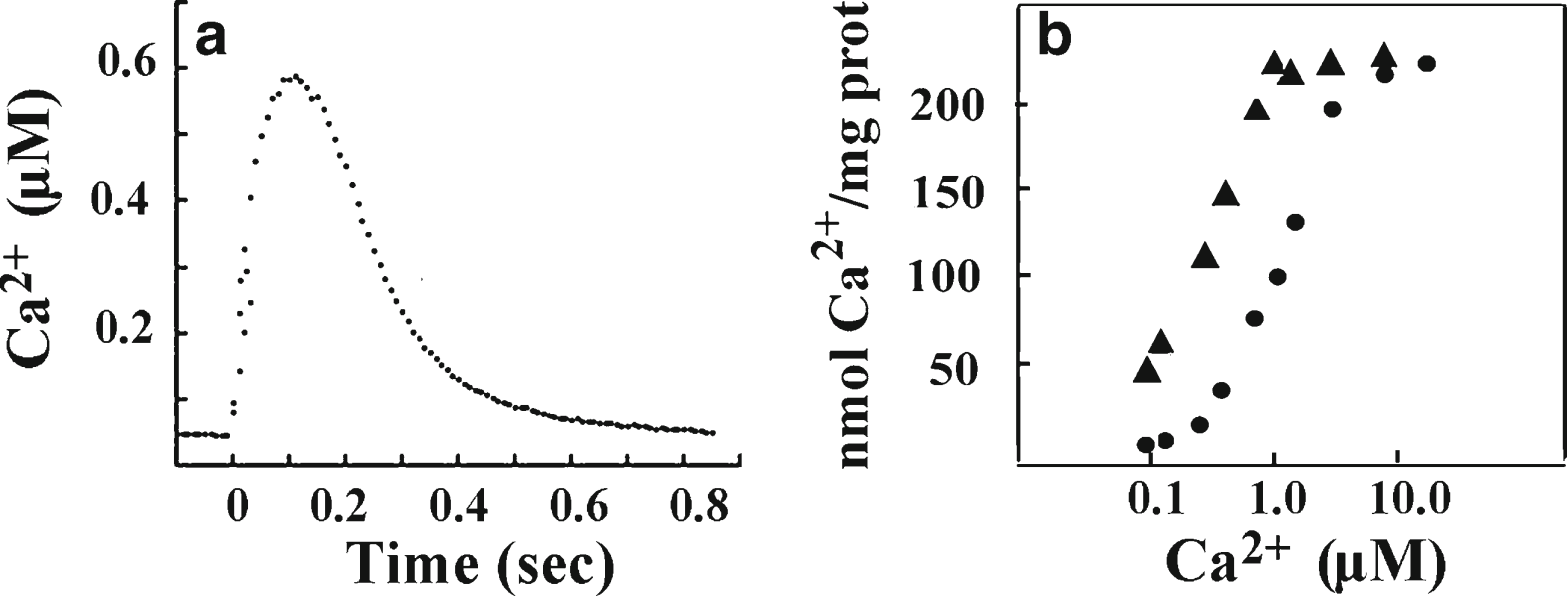
**a** Ca^2±^ signaling in cultured cardiac myocytes subjected to field stimulation. Following stimulation the cytosolic Ca^2+^ concentration rises from 0.04 μM to 0.6 μM, and then returns to the 0.04 μM resting level within 0.6 s. **b** Rates of Ca^2±^ uptake by cardiac sarcoplasmic reticulum vesicles as a function of free Ca^2±^ concentration. Before the measurements, the vesicles were pre-incubated with either a control buffer (*filled circle*), or with a monoclonal antibody neutralizing phospholamban (*filled triangele*). Note how neutralization of phospholamban decreases the Ca^2+^ concentration required for activation of the transport ATPase. Note also how the cytosolic Ca^2+^ concentrations observed at the low and high ends of the Ca^2+^ signal, correspond to Ca^2+^ levels indufficient or suitable to yield SERCA activation. Derived from Prasad and Inesi (2012) and Cantilina et al. (1993)

It is of interest that the Ca^2+^ ATPase SERCA2 isoform, prevalent in cardiac muscle (Lytton et al. 1992), is associated with phospholamban (PLN), a 52 amino acid polypeptide, to some extent similar to SLN. The definite effect of PLB on SERCA2 is a lower Ca^2+^ binding affinity (Koss and Kranias 1996; MacLennan and Kranias 2003; Toyoshima et al. 2003) and/or a slower E_1_ to 2Ca^2+^ · E_1_ transition (Cantilina et al. 1993). This results in a higher Ca^2+^ concentration requirement for Ca^2+^ transport activation (Fig. 6).

Both SLN and PLN reside within a groove surrounded by transmembrane helices M2, M4, M6 and M9, as shown by crystallographic studies (Toyoshima et al. 2013; Winther et al. 2013; Akin et al. 2013), and also indicated by NMR (Buffy et al. 2006) and cross-linking experiments (Sahoo et al. 2013). This is a critical position, since structural studies demonstrate that the helices delimiting this groove undergo displacements affecting Ca^2+^ binding, Ca^2+^ dissociation, as well as opening and closing of the lumenal gate. This explains how the presence of SLN and PLN may affect rates of movements and related partial reactions of the ATPase cycle. Some difference in the effects of the two polypeptides could be related to specific sequences and points of interactions (Sahoo et al. 2013), as well as to the cytosolic segment of PLN (absent in SLN) which may interact with SERCA headpiece domains and delay their movements to yield the E_1_^**.**^2Ca^2+^ state. It is worth considering that a slight shift of the Ca^2+^ concentration required for ATP activation would affect the rates of Ca^2+^ transport at low levels of cytoplasmic Ca^2+^, and therefore the efficiency of twitch relaxation. However, it is not likely that the Km (equilibrium constant) of the Ca^2+^ sites on lumenal orientation would be significantly affected. The physiological interest of PLB and SLN is related to the reversibility of their effects upon phosphorylation catalyzed by signaling kinases (Koss and Kranias 1996; MacLennan and Kranias 2003; Toyoshima et al. 2003).

## Contractile relaxation and uncoupling of the Ca^2+^ pump

Considering the possibility of Ca^2+^ pump uncoupling, an important question is whether uncoupling of SERCA1 interferes with reduction of cytosolic Ca^2+^ below the level allowing contractile relaxation of muscle fibers. The evidence presented above indicates that the Ca^2+^ pump is perfectly coupled when the SR lumen Ca^2+^_in_ is low, even if cytosolic Ca^2+^_out_ is relatively high. In the light of this information, we consider that relaxation of a muscle twitch occurs in less than 1 s and, within this time, lumenal Ca^2+^ does not reach a concentration higher than its dissociation from E_2_-P · 2Ca^2+^, as shown in experiments performed with rabbit native SR vesicles (Fig. 2). On the other hand, when cytosolic Ca^2+^ is reduced to a level producing contractile relaxation, such a Ca^2+^ level will be also low with regard to SERCA activation, and the ATPase would then proceed at very low rates or remain inactive. Therefore during relaxation, the pump remains quiescent, mostly in the Mg^2+^ bound E_1_ state (Toyoshima et al. 2013), with no significant slippage. Slippage of the pump would occur if lumenal Ca^2+^ were to become higher than its dissociation constant from E_2_-P · 2Ca^2+^, and cytosolic Ca^2+^ were to remain above the level required for full ATPase activation. This may occur upon prolonged muscle activity, if cytosolic Ca^2+^ is maintained relatively high by multiple action potentials and Ca^2+^ flux through plasma membrane voltage sensitive channels, as expected in shivering thermogenesis. Alternatively, a rise of intracellular pH above 7.0, may affect intracellular Ca^2+^ signaling, as recently reported for G protein signaling (Isom et al. 2013). In this case, some degree of SERCA uncoupling would be produced, contributing to the muscle twitching observed in alkalosis. Furthermore, thermogenic uncoupling may occur in tissues where SERCA is inserted in membrane compartments allowing lumenal Ca^2+^ rise to high levels, while cytosolic Ca^2+^ remains sufficiently high. Most importantly, it was reported that in brown fat, in addition to uncoupling of the mitochondrial respiratory chain, uncoupled SERCA contributes to non shivering thermogenesis (de Meis et al. 2006).

## Abbreviations

SERCA: Sarcoplasmic reticulum Ca^2+^ ATPase
SSM: Solid supported membrane
SR: Sarcoplasmic reticulum
SLN: Sarcolipin
PLN: Phospholamban
